# Polysaccharide-Based Drug Carriers—A Patent Analysis

**DOI:** 10.3390/gels10120801

**Published:** 2024-12-06

**Authors:** Snežana Ilić-Stojanović, Fouad Damiri, Adina Magdalena Musuc, Mohammed Berrada

**Affiliations:** 1 Faculty of Technology, University of Niš, Bulevar oslobodjenja 124, 16000 Leskovac, Serbia; 2 Laboratory of Biomolecules and Organic Synthesis (BIOSYNTHO), Department of Chemistry, Faculty of Sciences Ben M’Sick, University Hassan II of Casablanca, Casablanca 20000, Morocco; berrada_moh@hotmail.com; 3 “Ilie Murgulescu” Institute of Physical Chemistry, 202 Spl. Independentei, 060021 Bucharest, Romania

**Keywords:** fucoidan, pullulan, dextran, pectin, gels, drug delivery system

## Abstract

Polysaccharide-based carriers as biomaterials for drug delivery have been inspiring scientists for years due to their exceptional characteristics, such as nontoxicity, biocompatibility, and degradability, as they are able to protect pharmaceutically active molecules and provide their controlled/modified release. This review focuses on selected drug delivery systems based on natural polymers, namely fucoidan, pullulan, dextran, and pectin, with the aim of highlighting published patent documents. The information contained in patents is very important because it is usually not published in any other document and is much less discussed as the state of the art in the scientific literature. The Espacenet—European Patent Office database and the International Patent Classification were used for the research to highlight the specific search procedure. The presented analysis of the innovative state of the art includes an overview from the first patent applications to the latest granted patents in this field.

## 1. Introduction

Scientists in the world are conducting numerous studies in search of new drugs that would improve their effectiveness over those available in clinical practice. Many therapeutically active molecules that can be used in the treatment of specific diseases sometimes unexpectedly meet numerous obstructions and barriers in the human body that restrict or disqualify their administrations. One of the potential possibilities for improving therapeutic effects is the application of drug delivery systems [1]. Their usage enables the delivery of drugs/therapeutic molecules into the body and additionally enhances efficacy and safety by controlling/modifying their release rate and site of action [2,3]. The selected carrier in the drug delivery system has the main task of protecting the encapsulated drug/therapeutic molecule on the route from the application to the target diseased site and releasing it in a controlled way. For this purpose, carriers, such as “smart” polymers (hydrogels, nanogels) or colloids (liposomes), which chemically or physically bind the drug and release it as a response to biological stimuli (pH, temperature, pressure), have considerable progress [1,2,4]. However, there are certain limitations to their applications, such as the necessity of removing unreacted toxic synthetic reactants, low mechanical strength, and high production cost. Due to their natural availability, many advantages, and abundance, polysaccharides are currently very interesting in pharmaceutical applications [5]. Because of their inherent characteristics, such as nontoxicity, biocompatibility, biodegradability, flexibility, and renewability, they are the subject of considerable research. Polysaccharide-based carriers are able to protect pharmaceutically active molecules and provide their controlled/modified release [6]. An essential prerequisite for their pharmaceutical application is the characterization of the complex, fine structure of branched and backbone glycosidic fragments, which is possible by applying effective methods of their partial degradation [7,8]. Many scientists consider these degradation effects of complex polysaccharides, as well as the conformational relationships between the obtained fine molecular structures and their biological activity, with the aim of potential applications [9,10]. The composition and quantity of monosaccharides, as basic units, affect the biological activity of polysaccharides (such as anti-inflammatory, antioxidant, antitumor, immunomodulatory, antibacterial, and prebiotic). Modifications of structure, conformation, glycosidic bond type, molecular weight, and chain branching degree can improve or confer new biological activity of polysaccharides [11]. For example, various extracts obtained from Pleurotus species showed antitumor, antioxidant, antigenotoxic, immunomodulatory, antiplatelet-aggregating, anti-inflammatory, hypocholesterolemic, antihypertensive, antihyperglycemic, antimicrobial, and antiviral activities [12,13].

In addition to a large number of published scientific papers and reviews in this field, there is a lack of reports about their patentability. Therefore, the purpose of this review is to focus on state-of-the-art drug delivery systems based on fucoidan, pullulan, dextran, and pectin, highlighting published patent documents (especially granted rights) because the information contained in patents is usually not published in any other documents and is little discussed in the scientific literature [3]. The specific procedure of the search process in the patent database is also described. Patent documents not only contain unique information but also show innovative applications of polysaccharide-based drug delivery systems, research trends, industrial use, and clinical applications in current research and fill existing gaps in these areas. The presented review covered the period from the first patent applications to the most recently granted patents, which is specific for each analyzed polysaccharide (fucoidan, pullulan, dextran, and pectin).

## 2. Patent Search Methodology Analysis for Polysaccharide-Based Drug Carriers

The methodology for searching for appropriate patents was conducted on the “Espacenet” [14], a free database of the European Patent Office (EPO). The criteria for the search included classification symbols (using both the International Patent Classification (IPC) [15,16] and Cooperative Patent Classification (CPC) as a more detailed version of the ICP [17]) and definite keywords. Polysaccharides and derivatives thereof (e.g., starch, gums, dextrin, alginate, chitosan, hyaluronic acid, agar, inulin, or pectin), and their derivatives are classified in the A61K47/36 subgroup, based on IPC/CPC classification. Preparations with active medicinal ingredients are classified into subgroup A61K31/00, and the inactive modifying or targeting agents (i.e., inert additives, carriers) chemically bound to the active medicinal ingredient are classified into group A61K47/00. Medicinal preparation processes are classified into subgroup A61K9/00. The main selected groups and subgroups of IPC/CPC classification codes applied for this search are listed in Table 1.

First of all, patent search results were obtained using the IPC/CPC subgroup A61K47/36 for “polysaccharide” and keywords “gel” and “drug” and “carrier” or “delivery system” and “obtaining” or “procedure” or “method” or “process”) in the search field: full text or title, abstract, and claims.

**Figure 1:**
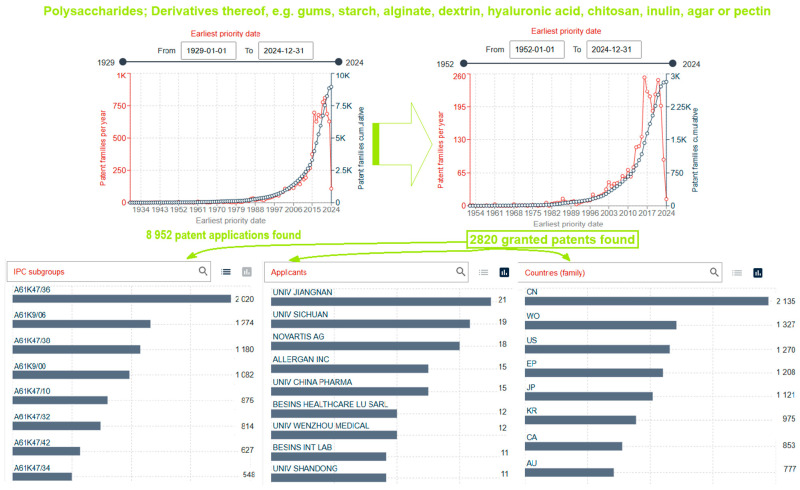
Drug carriers based on polysaccharide gels and derivatives thereof (e.g., gums, starch, alginate, dextrin, hyaluronic acid, chitosan, inulin, agar, or pectin): yearly and cumulative patent applications and granted patents numbers in dependence on the earliest priority date (from 1929 to 2024), the IPC subgroups, applicants, and countries for granted patents. Two-letter codes: CN, WO, US, EP, KR, CA, and AU are abbreviations for the following: Republic of China, the international publication of patent application using the Patent Cooperation Treaty (PCT) of the World Intellectual Property Organization (WIPO), United States of America, European Patent, Japan, Republic of Korea, Canada, and Australia, respectively. Data were obtained from the Espacenet database [14].

According to the mentioned criteria, 8952 patent documents (patent applications and granted patents) were found, of which 2820 were granted patent rights, in the period from the first patent application in 1929 to the most recent in October 2024. Figure 1 presents the graphical data of this search (patent applications and granted patents) depending on the earliest priority date and number of patents based on IPC classification codes, with data for the first nine applicants and countries with the largest number of granted patents.

## 3. Patent Analysis for Polysaccharide-Based Drug Carriers

### 3.1. Fucoidan-Based Drug Carriers

Fucoidan, an extract from the cell walls of brown algae (Phaeophyceae), such as *Fucus vesiculosus, Dictiota menstrualis, Kjellmaniella crassifolia,* and *Padina boriana* [18,19], kelps, large brown seaweeds, or algae (Laminariales) [20], belongs to the class of sulfated polysaccharides. It contains considerable amounts of fucose, various amounts of D-xylose, glucose, D-glucuronate, L-rhamnose, D-mannose, galactose, acetyl groups, and glucuronic acid. Fucoidan has various glycosidic linkages with different substituted sulfate and acetyl groups, as well as side branches containing other glycosyl units or fucose [21,22]. It was identified by Kylin more than 110 years ago [23]. Their structure and isolation and purification methods were studied in the following years [24,25], confirming the basic identification of this class of compounds. Different interests in fucoidan research dictate various extraction procedures. Aqueous systems are preferred because of the fucoidan water solubility [26]. Usually, extraction from some brown macroalgae includes multiple extractions, using hot hydrochloric acid and adding calcium to precipitate alginate during purification [11,27]. The methods of fucoidan extraction importantly influence the composition and yield [28]. Until now, no standardized method for fucoidan extraction has been available. Fucoidan was obtained using a conventional method of extraction from Irish brown seaweed, Fucus vesiculosus, and depolymerized by sonication [29]. Enzymes catalyzing partial cleavage of seaweed polysaccharides, for instance, fucoidanase and sulfatase, have been proposed to be useful tools for investigating the structural features and biological activity–function relationship of fucoidan polysaccharides [19].

**Figure 2:**
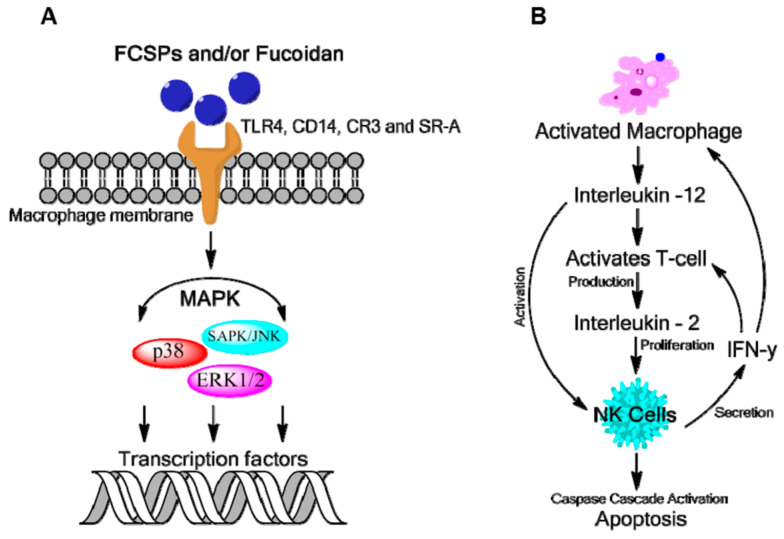
Proposed mechanism responsible for fucoidan bioactivity: (**A**) macrophage activation by fucose-containing sulfated polysaccharides (FCSPs) as mediated through specific membrane receptor activation, namely Toll-like receptor-4, cluster of differentiation 14, competent receptor-3, and scavenging receptor, which in turn induce intracellular signaling via mitogen-activated protein kinases (MAPKs); (**B**) activation of macrophages lead to the production of cytokines such as interleukins (ILs) IL-12 and IL-2 and interferon-gama (IFN-γ), which enhance natural killer (NK) cell activation that may stimulate T-cell activation further via IFN-γ. Reprinted from ref. [42] under open access creative commons CC-BY license.

Fucoidan activity has been investigated in many biological systems and is currently in research interest for its pharmacological uses all over the world. Its biological activities vary depending on species, structure, composition, molecular weight, and the method of administration. Their antitumor [18,19,30,31,32], strong antioxidant potential in vitro [18,31,33,34,35], antiviral activity [36,37], anti-angiogenic action [38,39], and immunomodulating activity [40] were recognized, and it is applied in the pharmacy and the food industry [41]. The mechanism responsible for the bioactivity of fucoidan is proposed in Figure 2 [42].

Fucoidan anticoagulant action is the most extensively investigated [43,44]. Many research studies point to biological effects after systemic delivery or oral administration, but little investigation has been performed about the uptake of fucoidan and its fate in metabolism [45]. Fucoidan from *Sargassum fulvellum* alleviates major metabolic disturbances, influences growth hormones and insulin-like peptides, and preserves intestinal homeostasis in larval and mouse models. There is increasing proof of the absorption of larger polysaccharide molecules into the body, even in small amounts [46]. Other studies present that degradation products of fucoidan with lower molecular weights are more attractive to oral development antithrombotic agents [47]. The anti-arthritis effect of fucoidan from brown seaweed was also investigated [48]. Interestingly, to achieve a therapeutic effect in vivo against crude snake venom, only high molecular weight fucoidan was effective, compared to lower molecular weight, which was ineffective.

The therapeutic potential of oral administration looks promising in various fields. Increasing the bioavailability and safety of fucoidan is important for the oral delivery of commercially available preparations in therapies, especially for new approaches to fibrosis and some neglected diseases.

**Figure 3:**
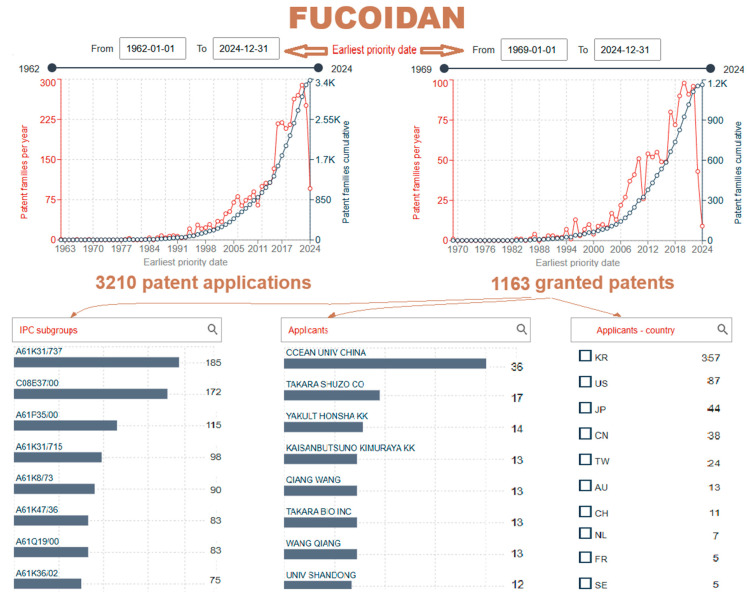
Fucoidan: cumulative and yearly numbers of patent applications and granted patents depending on the earliest priority date (from 1962 to 2024), the IPC and CPC subgroups, the first eight applicants, and countries for granted patents. Two-letter codes: KR, US, JP, CN, TW, AU, CH, NL, FR, and SE are abbreviations for the following: the Republic of Korea, United States of America, Japan, Republic of China, Taiwan Province of China, Australia, Switzerland, Netherland, France, and Sweden, respectively. Data were obtained using the Espacenet database [14].

In the Espacenet database [14], a search shows that there are 3210 patent documents and 1163 granted patents in the period from the first patent application in 1962 to the latest on 20 October 2024 for “fucoidan”, according to the patent claims (Figure 3). The country of origin of the first granted patent JPS505199B1 was Japan [49]. Based on IPC/CPC subclasses, the largest number of granted patents is classified into the following classes:

- A61K31/737—Sulfated polysaccharides (185);
- C08B37/00—Preparation of polysaccharides (172);
- A61P35/00—Applied as antineoplastic agents (115);
- A61K31/715—Polysaccharides, i.e., having more than five saccharide radicals attached to each other by glycosidic linkages; derivatives thereof, e.g., ethers, esters (98);
- A61K8/73—Cosmetics or similar toiletry preparations containing polysaccharides, medicinal preparations characterized by the non-active ingredients used, e.g., carriers or inert additives (90);
- A61K47/36—Targeting or modifying agents chemically bound to the active ingredient contained polysaccharides; derivatives thereof, e.g., gums, starch, alginate, dextrin, hyaluronic acid, chitosan, inulin, agar, or pectin (83);
- A61Q19/00—Preparations for care of the skin (83);
- A61K36/02—Medicinal preparations of undetermined constitution containing material from algae or derivatives thereof, e.g., traditional herbal medicines (75).

From the year 2015, the number of patents extensively grew yearly. The patent search was additionally narrowed by adding keywords for specific fields, e.g., “fucoidan”, “gel”, “drug”, “carrier”, “deliver”, “obtaining”, “procedure”, “method”, “process”, “extract”, and “administration” only for granted patents. Using selected keywords, a total of 59 patents were granted after 1995, from 213 patent applications (with earliest priority data from 5 April 1991). After their visualization, selected granted patents relevant to this topic are summarized in Table 2 and later analyzed.

The anti-wrinkle microemulsion gel eye patch, according to the CN118319827B invention, includes photoreactive functional ingredients: natural plant extract (*Elsholtzia jiangensis, Galla chinensis,* and semen *Ziziphi spinosum*) with higher antioxidant performance and elastase inhibition performance, and a marine extract based on 4–6% of fucoidan solution [50].

**Figure 4:**
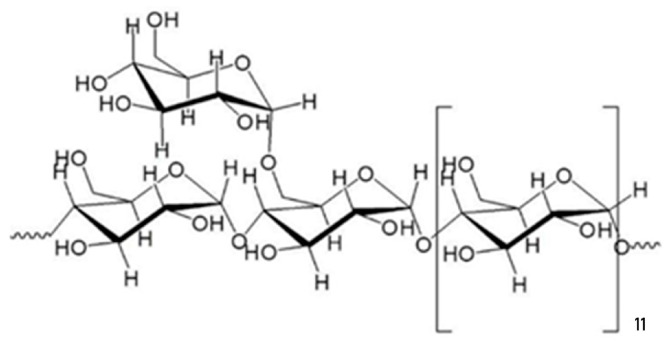
Structural formula of fucoidan homopolysaccharide from brown algae *Sargassum*, according to data presented in granted patent CN117247472B [51].

Granted patent CN117247472B [51] discloses a new fucoidan homopolysaccharide from brown algae *Sargassum*, with a structural formula shown in Figure 4 with an average molecular weight of 27,476 g/mol and the number-average molecular weight of 20,588 g/mol, formed by polymerizing D-glucose and α-d-Glcp-(1→,→4)-α-d-Glcp-(1, and →4,6)-α-d-Glcp-, the molar ratios are 5:87.5:7.5, respectively). This patent described the method of obtaining it and its application for the prevention/treatment of osteoporosis. The fucoidan (12.5–50 μg/mL) was used in the drug preparation to promote cell osteogenic differentiation and inhibit adipogenesis.

The objective of CN116077417B patent is a gastric retention tablet comprising 10–30% functional ingredients, 10–30% filler, 30–50% hydrophilic gel material (fucoidan and hydroxypropyl methylcellulose), and 4–10% foaming agents [52]. The weight ratio of fucoidan to hypromellose in the hydrophilic gel is 1:1.5–1:4 (the amount of hypromellose is not less than 25%).

Microbeads for embolization consisting of biocompatible polymer (cross-linked gelatin) and iron adsorption block (containing fucoidan and/or polyphenol) for the treatment of proliferative diseases are protected by granted patent KR102386631B1 [53]. They can adsorb iron and effectively block its transfer to cancer cells in an embolization process, which has greatly improved the efficacy of the cancer treatment (liver cancer).

The invention CN112999361B discloses a CuET@Fuc nano-drug delivery system with the antitumor effect of the inner core disulfiram copper complex, associated with the fucoidan shell, modified on the inner core disulfiram copper complex [54]. The tumor treatment effect is greatly enhanced by the accumulation of the disulfiram copper nanocrystal at a tumor part and the targeting of P-selectin.

Anticoagulant fucoidan oligosaccharide, described in patent CN112521431B, was characterized by a high-purity disulfated fucotriose, and obvious anticoagulant activity, which can importantly prolong apoptosis compared to the state of art and its preparation method [55]. The brown alga used for this invention is hijiki (a black fibrous seaweed). Obtained anticoagulant fucoidan oligosaccharide, according to this patent, was used in preparing anticoagulant medicine and/or health food.

Fucoidan composition and application thereof for treating fibrosis of a target muscle (striated muscle, smooth muscle, and cardiac muscle) were protected by patent TWI747567B [56]. This composition includes a cross-linked first polymer (the material system is composed of polyol and fucoidan) water and a second polymer (hyaluronic acid or sodium alginate). The composition contains 0.1–10% of the fucoidan, with 10–100% fucose and 20–50% sulfate, wherein the ratio of fucose and sulfate in the fucoidan is 1:5–4:1.

The invention CN111888430B [57] protects composite antibacterial gel for an auxiliary treatment effect on pruritus, abnormal leucorrhea, and pudendum odor (caused by bacterial cervicitis and vaginitis). It comprised of several components: tea tree essential oil (36–44 parts), lemongrass essential oil (18–22 parts), *Clausena lansium* pectin (70–80 parts), fucoidan (6–10 parts), carbomer (10–14 parts), polyoxyethylene 40 hydrogenated castor oil (180–220 parts), and polyhydric alcohol (330–400 parts). This antibacterial gel has an inhibiting effect on *Staphylococcus aureus*, *Escherichia coli*, *Candida albicans,* and *Candida glabrata*.

A nano-targeted drug delivery system for co-transport of thrombolytic and antiplatelet drugs is described in patent CN111840527B [58]. A method of preparation of a shear-responsive nano-drug delivery system based on β-cyclodextrin and adamantane is grafting onto poly(lactic-co-glycolic acid) and fucoidan molecular skeletons. The fucoidan supramolecular self-assembled nanoparticles are prepared with β-cyclodextrin vesicles. In the process of mediating self-assembly to form a shear-responsive drug release, urokinase is encapsulated in the network of polysaccharide nanoparticles.

Hemostatic composition in the gel (pasty) form, which contains chitosan and fucoidan, 1:(0.5–10)/w:w, preparation method, and its application were protected by patent CN111760066B [59]. The purity of the fucoidan sulfate is ≥95%. This gel is suitable for on-site first-aid hemostasis of deep, massive bleeding, which cannot be efficiently filled using conventional hemostatic dressings. The hemostatic gel composition with better plasticity and fluidity better fills gaps and seals wound surfaces in non-compressible bleeding (various complex irregular wounds, groins, armpits, etc.) and quickly stops bleeding.

A method for mesenchymal stem cells differentiation from pluripotent stem cells was presented at invention KR102275454B1 [60]. Used adhesive polymers are hyaluronic acid, alginic acid, heparin, fucoidan, cellulose, dextran, chitosan, albumin, fibrin, collagen, and gelatin. Obtained mesenchymal stem cells have an excellent proliferation rate and a high yield of superior intrinsic biological activity by sequentially performing three-dimensional suspension culture. Produced mesenchymal stem cells show a high differentiation efficiency into cartilage and bone with excellent anti-inflammatory activity and can be used as a composition for treatments of bone or cartilage diseases, inflammation, autoimmune diseases, and similar.

The method of preparation of fucoidan-coated gated mesoporous manganese dioxide nano-drug-loading system and its application are objects of granted patent CN111298133B [61]. Nanoparticles of mesoporous manganese dioxide can catalyze hydrogen peroxide to produce oxygen. As a matrix material, the fibrinolytic drug urokinase was applied as a model drug, and fucoidan was modified on the surface of manganese dioxide through thrombin degradable peptides to construct a nano-drug delivery system with thrombus targeting and thrombin-responsive drug release to relieve Hypoxia at the thrombus site.

Granted patent US11540980B2 protects personal care compositions for generating increased foam volume and obtaining methods [62]. This composition contains a carrier and an effective amount of at least two hydrocolloids, e.g., xanthan gum and sulfated polysaccharide, in a ratio of about 1.5:1–9:1. The first hydrocolloid consists of sulfated polysaccharides (fucoidan, carrageenan, chondroitin sulfate, keratan sulfate, funoran, dextran sulfate, dermatan sulfate, porphyrin, heparin, and combinations thereof).

**Figure 5:**
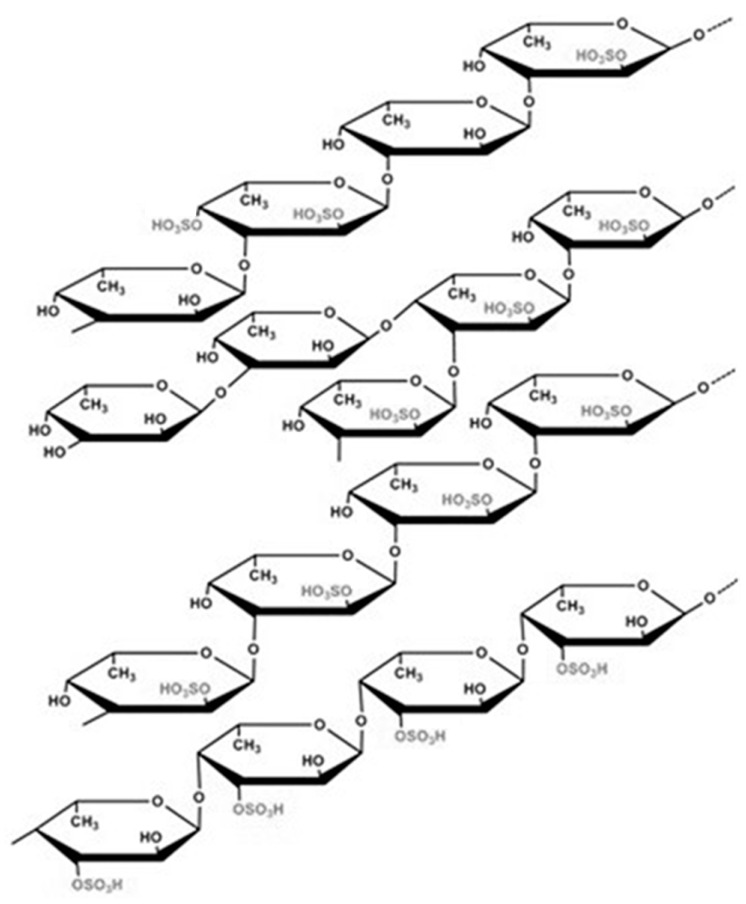
The main structure of the sea cucumber fucoidan, derived from Aegean sea cucumber *Holothuria polii*, according to data presented in granted patent CN110437288B [63].

The invention CN110437288B discloses a novel sea cucumber fucoidan derived from Aegean sea cucumber *Holothuria polii* for the first time, its method of preparation, and its application [63]. The mass ratio of fucose in the sea cucumber fucoidan is 95.7%. The novel sea cucumber fucoidan has a weight-average molecular weight of 150–200 kDa, a sulfate mass ratio of 30–45%, a tetrasaccharide repeating unit composed of α-1→3-linked fucose, wherein the repeating unit is [→3-α-l-Fucp-1→3-α-l-Fucp2(OSO_3_−)-1→3-α-l-Fucp2(OSO_3_−)-1→3-α-l-Fucp2,4(OSO_3_−)-1→], presented as the structural formula in Figure 5.

The sea cucumber fucoidan may restore the reduction of leukocytes and neutrophils induced by cyclophosphamide. The fucoidan has the effect of stimulating hematopoiesis and good endogenous anticoagulant activity.

The objective of granted patent CN110101671B is a tablet based on voglibose, an α-glucosidase inhibitor, wherein xanthan gum and/or fucoidan are the macromolecular polysaccharide included with 10–20 parts in the final formulation [64].

The preparation method of the Sargassum henslowianum fucosan sulfate composed of fucose and galactose (molecular weight of 4.0 × 105 to 6.0 × 105) extracted from Sargassum henslowianum and its application were disclosed in granted patent CN111748045B [65]. The fucose amount in the Sargassum heinz fucoidan sulfate is 74–77%. The Sargassum henslowianum fucosan sulfate may have an antiviral role as an effective inhibitor of herpes viruses’ adsorption on the host cells’ surface. It can act on the cell´s surface during virus infection without entering the cells, so the possibility of toxic and side effects is quite small.

Disclosure GB2595109B relates to pharmaceutical delivery compositions containing natural products (e.g., extracts) combined with biotechnological excipient systems, biosurfactants suitable for the repair of compromised skin (e.g., oral mucosa tissue), and their use for skin repair and regeneration [66]. The composition comprises (i) a drug delivery system comprising a carbonate buffer solution, at least two different biosurfactants, at least one hygroscopic agent, and at least one antioxidant and (ii) at least one bioactive agent. This composition has a pH value of about 7.5–9.5. The bioactive natural extract comprises one or more sulfated polysaccharides selected from carrageenan, ulvan, galactan, and fucoidan.

Granted patents AU2019310194B2 [67] AU2019310360B2 [68] and ZA202100275B [69] protect medically acceptable compositions, methods, and systems based on highly purified fucans with low endotoxin applied for the fibrous adhesion treatments. Highly purified and modified fucans and their compositions have a reduced level of undesired components, impurities, or non-fucan components found in the initial composition. A fucan low-endotoxin composition contains less than about 0.2–0.0005 endotoxin units/mg of the fucan. Fucan used in the composition has a weight-average molecular weight of about 100–500 kDa and a sulfation level of about 20%–60% *w*/*w*. Low-endotoxin compositions of fucan are suitable for medical/surgical uses (e.g., prevention, inhibition, reduction, removal, or the other treatment of fibrous adhesions). These compositions decrease risky complications of endotoxins (inflammation, fever, and endotoxemia) through the medical/surgical application of fucans. The target site could be an abdominal cavity, a pelvic cavity, a dorsal cavity, a spinal cavity, a cranial cavity, a ventral cavity, a pleural cavity, a thoracic cavity, a pericardial cavity, a joint, skin, a tendon, a muscle, and a ligament.

According to patent KR101973806B1 [70], a core–shell nanofiberPCL@gelatin/fucoidan was prepared using hydrophobic (polycaprolactone (PCL) and 2,2,2-trifluoroethanol) and hydrophilic polymer solutions (gelatin, fucoidan, 2,2,2-trifluoroethanol and distilled water), fabricated by electrospinning, and characterized.

Biocompatible nanoparticles produced by electron beam irradiation into an aqueous solution, according to patent KR101893549B1 [71], contained at least one polysaccharide (fucoidan, α-cyclodextrin, β-cyclodextrin, γ-cyclodextrin, mannan, fructo-oligosaccharides, inulin, isomalto-oligosaccharides, carboxymethyl dextran, glycogen, amylose, beta-glucan, and chondroitin). The product can be used as a drug carrier, a diagnostic agent, a contrast agent, or a means to prevent intestinal adhesions or to prevent and treat disease.

Patent TWI612978B showed oral dissolving film with rapid action onset, increased drug availability, full therapeutic effects, and reduced side effects [72]. It comprises first thickener, 0.1–5 wt% (sodium carboxymethyl cellulose, methyl cellulose, hydroxyethyl cellulose, modified starch, sodium polyacrylate or polypyridyl pyrrolidone), second thickener, 0.1–10 wt% (fucoidan or red algae (furcellaran), sodium caseinate, gelatin, chitosan, chitin, whey protein isolate, insulins, whey protein concentrate, condensation zymosan, polysaccharide, alginic acid (salt), propylene glycol alginate), gelling agent, 0.1–10 wt% (locust bean gum, Tamarind gum, flaxseed gum, *acacia beans* (honey locust gum), Indian gum, karaya gum, peach gum, pectin, konjac glucomannan, *Indian aloe* extract, insythrine or mesona chinensis polysaccharide), and the water up to 100%.

According to patent CN103804504B [73], the process for the preparation of low molecular fucoidan sulfate includes the following steps: (1) acid degradation of fucoidan sulfate with hydrochloric acid (0.05–0.2 mol/L) at 50–60 °C, during 12–18 h; (2) coarsening using an ultrafiltration membrane with a molecular weight cut off of 6000 Da; (3) obtained low molecular fucoidan sulfate was desalted by Sephdex G-10 gel column and separated by Q-Sepharose ion exchange column. This product acts on diabetic nephropathy and can significantly inhibit the proliferation of mesangial cells and inhibit the excessive secretion of TGF-β1 and Col-excessive expression of IV.

Patent KR101239782B1 [74] protects the scaffold containing a drug delivery system and the obtaining method. The cell carrier consists of the biocompatible natural polymer (collagen, alginate, fucoidan, or chitosan) with the addition of hydroxyapatite or beta-tricalcium phosphate.

The analyzed granted patents included new structures of fucoidan, new methods of obtaining it, and its application in the prevention/treatment of osteoporosis, cancer, and muscle fibrosis. It has been found to have application within various compositions with anticoagulant, antineoplastic, antiviral, antibacterial, and hemostatic effects. Granted patents disclose its application as a drug carrier or inert additive; it has been used as an adhesive polymer for the differentiation of mesenchymal stem cells. It has also been applied to preparations for the care, repair, and regeneration of damaged skin as a hydrophilic gel material, a contrast agent, and a diagnostic agent for the prevention and treatment of intestinal diseases.

### 3.2. Dextran-Based Drug Carriers

According to the IUPAC definition, dextran is a branched poly-α-D-glucoside of microbial origin having glycosidic bonds predominantly C-1 → C-6 [75]. Precisely, it consists of linear D-glucose chains, linked by α-(1→6) glucopyranoside units, with probable branches with D-glucose, which are linked by α-(1→2) bonds or α-(1→4), α-(1→3) branched chains, with molecular weights ≥1000 Dalton, and could be with high or low molecular weight (>40 kDa or <40 kDa, respectively) [76,77]. The degree and nature of the branching units at positions 2, 3, and/or 4 are determined by the dextran-producing bacterial strain. Historically, Louis Pasteur discovered dextran in wine as a product of the slime-producing bacteria *Leuconostoc mesenteroides* [78]. Further investigations have shown that dextran can be produced from various Gram-positive, facultatively anaerobic cocci (*Leuconostoc, Weissella*, *Lactobacillus*, and *Streptococcus*) [66,79]. Dextran, as an exopolysaccharide, can be synthesized by lactic acid bacteria or their enzymes in the presence of sucrose [80]. Diaz-Montes recently compiled a review on synthesized high- or low-molecular-weight dextran using modified enzymes and unconventional sources, as well as using lactic acid bacteria for the production of dextran with specific structural characteristics [66]. Dextran clinical grade (40, 60, and 70 kDa molecular weight) in aqueous solutions (6 and 10%) is available as blood plasma substitutes [81].

**Figure 6:**
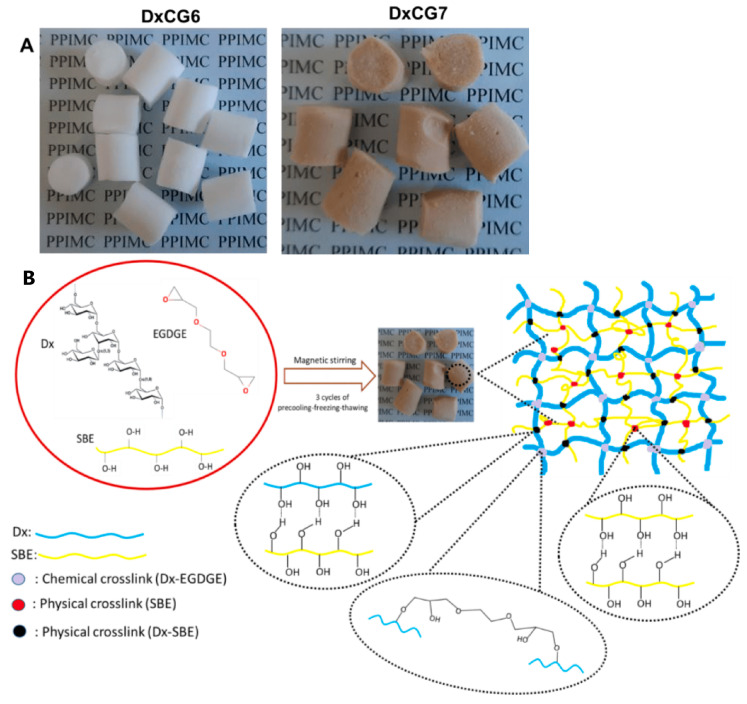
(**A**) Optical images with dextran (Dx)-based cryogels prepared without SBE (sample DxCG6) and with SBE (sample DxCG7); (**B**) schematic representation of the Dx cross-linking with EGDGE and the interactions through hydrogen bonding between SBE and Dx matrix. Reprinted from ref. [87] under open access creative commons CC-BY license.

Dextran, as a carrier for controlled/modified drug delivery, is usually used due to its biological compatibility, degradability, and chemical modification possibility. The human body tolerates dextran well as a natural exopolysaccharide, with a reduced risk of immune reactions, which makes it suitable for clinical/medical applications. It can be easily chemically modified/functionalized through hydroxyl functional groups to improve control of drug release, and it can be conjugated with different active substances for targeted delivery (e.g., gels, hydrogels, microspheres, micelles, nanostructures) [82,83]. Dextrans are soluble because of their ability to form hydrogels and incorporate large amounts of water. The application of dextran hydrogels is an excellent strategy, and it is the center of much research attention. A few hydrogels were developed using dextrans (70 and 148 kDa) and polymer blends in various proportions with the poorly water-soluble drug praziquantel to evaluate the solubility enhancement from polymeric delivery systems [84]. Magnetic/pH dual responsive dextran hydrogels were applied as stimuli-sensitive carriers for anticancer drug doxorubicin with diffusion-controlled mechanism, swelling, and simultaneously erosion processes [85]. An injectable and conductive gelatin methacrylate/oxidized dextran hydrogel with incorporated umbilical cord mesenchymal stem cells was studied by Zhu and colleagues. This dextran-based hydrogel could improve and reconstruct damaged myocardial tissue and could be a promising therapeutic strategy for myocardial infarction treatment [86]. Porous hydrogels based on dextran and the encapsulation of natural extract from *Picea abies* spruce bark within the dextran network were successfully synthesized using a dual cross-linking method, which included chemical cross-linking and physical stabilization by freeze–thawing [87]. Obtained hydrogels (Figure 6) possess antioxidant and antimicrobial properties and provide new possible applications in biomedical fields.

The dextran-based hydrogel was changed by grafting using poly(ε-caprolactone) and cross-linked by polyethylene glycol 400 to yield a gel with covalent cross-linking and improved mechanical characteristics. Adipose-derived stem cells proliferated well after seeding in it and formed micro-mass. In vitro and in vivo angiogenesis and in vitro osteogenesis demonstrated the potential of an obtained hydrogel system for vascularized bone tissue engineering [88]. Numerous studies and published papers confirm the wide usage of dextran hydrogels as constituents for biomedical applications, including biosensor materials, drug delivery devices, and tissue engineering scaffolds.

**Figure 7:**
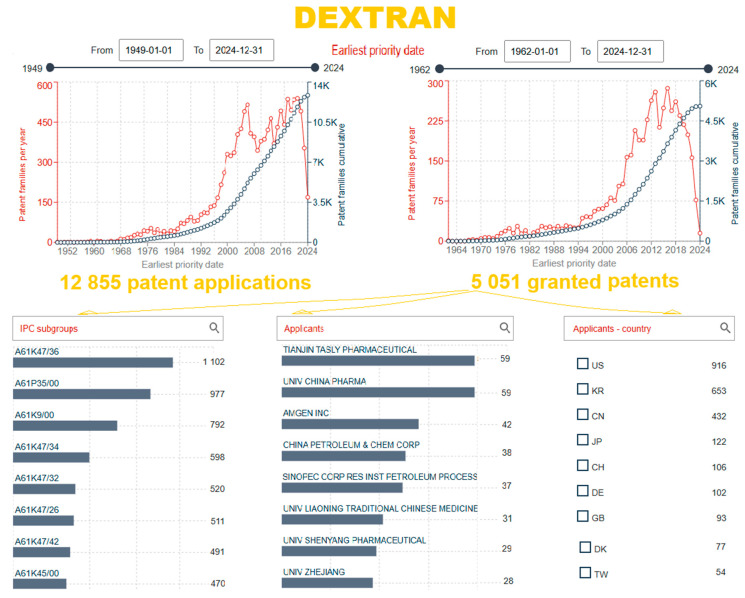
Dextran: cumulative and yearly numbers of patent applications and granted patents depending on the earliest priority date (from 1949 to 2024), the IPC subgroups, the first eight applicants, and countries for granted patents. Two-letter codes: US, KR, CN, JP, CH, DE, GB, DK, and TW are abbreviations for the following: United States of America, Republic of Korea, Republic of China, Japan, Switzerland, Germany, Great Britain, Denmark, and Taiwan Province of China, respectively. Data were obtained using the Espacenet database [14].

In the Espacenet database [14], a search shows that there are 15251 patent documents and 5051 granted patents from the first patent application in 1962 to the most recent on 20 October 2024 for “dextran”, according to the patent claims (Figure 7). The country of origin of the first granted patent, DE1168605B, was Germany [89]. Based on IPC/CPC subclasses, the largest number of registered patents is classified into the following classes:

- A61K47/36—Polysaccharides; derivatives thereof, e.g., dextrin (1102);
- A61P35/00—Applied as antineoplastic agents (977);
- A61K31/737—Sulfated polysaccharides (185);
- A61K9/00—Medicinal preparations characterized by special physical form (792);
- A61K47/32—Macromolecular compounds obtained by reactions only involving carbon-to-carbon unsaturated bonds (520);
- A61K47/34—Macromolecular compounds obtained other than by reactions only involving carbon-to-carbon unsaturated bonds (598);
- A61K47/26—Medicinal preparations characterized by the non-active ingredients used, e.g., carriers or inert additives; targeting or modifying agents chemically bound to the active ingredient, organic compounds, carbohydrates, e.g., sugar alcohols, amino sugars, nucleic acids, mono-, di-, or oligo-saccharides; derivatives thereof, e.g., polysorbates, sorbitan fatty acid esters or glycyrrhizin (511).

Although the development of technology and application of dextran started intensively in 1949, a trend of expansive development and patenting has been observed since 2008. The patent search was additionally narrowed by adding keywords for specific fields, e.g., “dextran” and ”gel” in the field claims, “gel”, “drug”, “carrier”, “deliver”, “obtaining”, “procedure”, “method”, “process”, “extract”, and “administration” only for granted patents. Using the selected criteria, a total of 458 patents were granted after 1970 from 932 patent applications. Following their visualization, the selected relevant granted patents were summarized in Table 3 and subsequently analyzed.

According to patent CN115594776B [90], a reactive oxygen species (ROS)-responsive polymer maleimide-modified PHB-Dextran (molecular weight of 10,000–25,000) was applied as a self-assembly nanovesicle drug delivery carrier. The method of the cell knapsack system preparation is simple. In the nanovesicle carrier, an aqueous surfactant solution (0.4 to 0.6%) could be poloxamer, alkylphenol polyoxyethylene ether, and polyoxyethylene amide. ROS-responsive polymer PHB-Dextran can be combined under a mild condition with a drug or a cell by a thiolene click reaction and cell knapsack drug delivery system. The obtained system is suitable for carrying vaccines, drugs, nano-therapeutic substances, and imaging agents, for targeted delivery to areas associated with inflammation in the central nervous system, and for acute/chronic inflammatory diseases (caused by biological pathogens) and immune-mediated inflammatory demyelinating diseases.

The invention CN112876578B [91] provides self-assembled nanoparticles of amphiphilic dextran derivative carriers for targeting tumor-associated fibroblasts and its pharmaceutical application. This carrier includes a hydrophilic dextran-enhanced derivative skeleton, a hydrophobic group molecule, and a linker arm of FAP-α responsive peptide fragment. After the nanoparticles reach a focus part, a specific peptide fragment connecting the arm can be subjected to enzyme digestion by FAP-α, specifically and highly expressed on the surface of tumor-associated fibroblasts when the nanoparticles are rapidly degraded. Physically loaded drugs can quickly be released from these disintegrated nanoparticles, and tumor-associated fibroblasts can be effectively inhibited or killed. The physical barrier of a tumor matrix can be weakened, and drug permeation can be enhanced.

The invention disclosed in patent CN106978410B [92] comprises difunctional dextranase with the chitosan hydrolytic activity, an engineering strain, a carrier, a gene, and the difunctional dextranase application in the chitosan degrading process to produce oligosaccharide of chitosan. The new high-temperature-resistance difunctional dextranase was disclosed by the invention with excellent heat resistance, optimum action temperature of 55 DEG C, stability in enzyme activity under pH of 2.0 to 6.5, can efficiently hydrolyze high concentrations of chitosan with 100 g/L, and does not generate a monosaccharide byproduct. The enzyme activity can reach 1000 U/mL, which is much higher than a common production level.

The invention CN106860875B [93] protects a preparing method for antitumor non-pH-sensitive and pH-sensitive dextran–polylactic acid–polyethylene glycol nanoparticles. Polyethylene glycol is used as the initial material, followed by ring-opening polymerization with D,L-lactide (lactic acid) to form a polylactic acid–polyethylene glycol polymer, which reacted with butanedioic anhydride to obtain a polylactic acid–polyethylene glycol polymer with carboxyl groups (mPEG-PLA-COOH). An esterification reaction with dextran is performed to obtain a dextran–polylactic acid–polyethylene glycol carrier (Dextran-PEG-PLA). Based on this carrier, the polymer is further reacted with methoxypropene and propionic anhydride, yielding both non-pH-sensitive (propionic-dextran-PEG-PLA (PDP)) nano-carriers and pH-sensitive (acetalated-dextran-PEG-PLA (ADP)). The preparation method for these nano-carriers is easy, the raw materials are inexpensive and accessible, and these nano-carriers are appropriate for clinical drug applications.

Granted patent TWI644687B [94] disclosed dextran magnetic iron nanoparticle, which comprises an active drug suitable for cancer treatment and for imaging preparation, obtaining method, and use for cancer treatment. This is a single-crystalline-layer dextran magnetic iron nanoparticle, coated outside with a dextran thin layer (thickness less than 3 nm) with a diameter less than 50 nm. The active drug contains amino groups (e.g., erlotinib) that are bonded with the dextran layer by non-covalent bonds. Dextran magnetic iron nanoparticles can be moved by an applied magnetic field and stagnate at the cancer site.

The invention CN106421805B [95] discloses a dextran cross-linked hemoglobin-based oxygen carrier, its preparation method, and its application. Each hemoglobin tetramer in the oxygen carrier comprises two thiol groups, protected and mixed with activated dextran until cross-linking with the bovine hemoglobin to give a dextran cross-linked hemoglobin oxygen carrier. The activated dextran is obtained by oxidizing a hydroxyl group on dextran 40 to an aldehyde group using sodium periodate. This carrier can be used for the preparation of a blood substitute in the process of blood transfusion. It partially/fully replaces red blood cells in a carrying/releasing function when the oxygen saturation is 50% when the blood transfusion process is caused by blood loss, trauma, surgery, or anemia.

The invention KR101842059B1 [96] relates to a photo-crosslinkable dextran polymer drug delivery agent, introduced with an anionic group and an acrylate group, parallel with a molding process. The production method is very simple compared to the conventional method, and it is easy to control a composition ratio arbitrarily. This product can be used as a drug delivery agent. It is biocompatible by using biodegradable dextran, which induces interaction between the anionic functional group and the drug, and thus, stable deposition and sustained release of the drug can be achieved.

An object of patent CN105148279B [97] refers to the dextran nano-drug carrier, its preparation method, and antitumor drug. The dextran nano-drug carrier contains carboxylated dextran nanoparticles, grain size 20–100 nm. They have good biological inclusivity and degradability and can load drugs. Due to the structural characteristics of carboxylated dextran, the drugs can be loaded via hydrazone bonds that are pH-sensitive (potential of hydrogen) and break down in a specific pH environment. Therefore, the dextran nano-drug carrier can be used to establish a pH-responsive drug release system so that a curative effect is improved and side effects are reduced.

Granted patent EP2921166B1 [98] disclosed biodegradable microbeads containing albumin and dextran sulfate with improved anticancer drug adsorptivity and its preparation method. The present invention can be useful for liver cancer chemoembolization. The microbeads are prepared from a biocompatible and biodegradable polymer to be safe for the human body. They effectively inhibit the growth of a tumor by continuously releasing an anticancer drug adsorbed on the surfaces of the beads and effectively blocking a blood vessel that supplies nutrition to a liver tumor.

The benefit agent delivery particles composition comprising dextran (molecular weight above 5 kD–20 kD) as a delivery aid, which may include a non-polysaccharide, aminoplast polymer, and a perfume, is provided by invention EP2747741B1 [99]. The invention also provides a manufacturing process for the particles in which perfume oil is encapsulated by emulsion polymerization to form core–shell particles. Alternatively, the perfume may be adsorbed later. This composition comprises an enzyme selected from hemicellulase, cellulase, xylanase, polygalacturonase, pectinase, pectate lyase, mannanase, ligninase, pentosanase, pullulanase, hyaluronidase, arabinosidase, laccase, chondroitinase, amylases, glycosylhydrolase, and/or mixtures thereof.

Most of the analyzed granted patents on dextran gels cover drug preparations of special physical forms with inactive ingredients or inert additives, e.g., self-assembling nanovesicle carriers, suitable for targeted delivery of drugs, vaccines, nanotherapeutics, imaging agents, to areas associated with inflammation or tumor. Targeting or modifying agents are chemically bound to the active ingredient, organic compounds, and carbohydrates. Dextrans were applied as cross-linked hemoglobin-based oxygen carriers as antineoplastic agents. They are suitable for clinical drug delivery and are used to establish a pH-sensitive drug release system, thereby reducing side effects and improving the curative effect.

### 3.3. Pullulan-Based Drug Carriers

**Figure 8:**
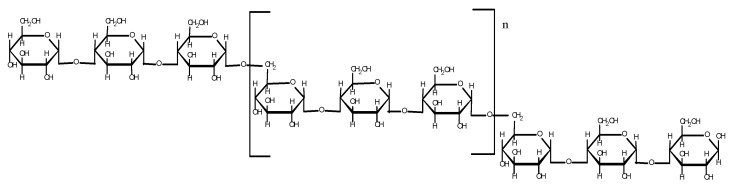
The structural unit of pullulan (maltotriose (α-1,4-triglucoside) interconnected by α-(1→6) glycosidic bonds), according to data presented in granted patent EP1363951B1 [100].

Pullulan is a neutral amorphous, water-soluble, viscous polysaccharide with linear, inflected chains whose main axis consists of the units α-D-glucose, firstly formed by the activity of *Aureobasidium pullulans* fungi at starch. A regularly repeated structural unit in pullulan is maltotriose (α-1,4-triglucoside) interconnected by α-(1→6) glycosidic bonds, shown by the structural formula in Figure 8 [100].

**Figure 9:**
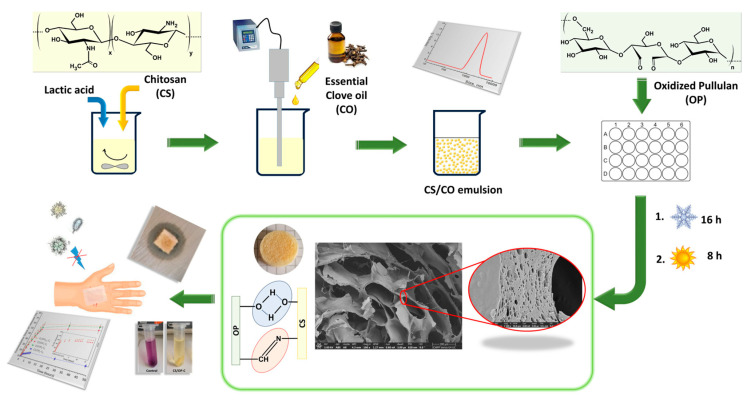
Chitosan-oxidized pullulan hydrogels loaded with essential clove oil: synthesis, characterization, antioxidant, and antimicrobial properties—graphical abstract. Reprinted from ref. [111] under open access creative commons CC-BY license.

It was revealed by Bauer in 1938 [101] and called “pullulans” by Bender in 1959 [102]. Some fungal species (*Aureobasidium* spp., *Cytaria* spp., *Teloschistes flavicans*, *Rhodototula bacarum*, and *Cryphonectria parasitica*) have been used for commercial production, but *Aureobasidium pullulans* is preferred due to superior properties of produced pullulan and high production rate [103]. Nowadays, the current interest is pullulan production from low-cost substrates to reduce its price. Pullulan is a non-branched, non-immunogenic, non-toxic, non-carcinogenic, non-mutagenic exopolysaccharide explored for various pharmaceutical (e.g., for targeted hydrophobic and hydrophilic drugs or gene delivery, tissue engineering, wound healing [104]) and medical applications (e.g., in diagnostic usage like receptor, perfusion and lymph node, target-specific imaging, and vascular imaging) [105,106]. However, these poor mechanical features are improved by combining them with various polymers. Pullulan is used to design hydrogels, with chemical modification [107,108], to improve some biological, physical, and chemical properties for potential medical applications [109]. The incorporation of pullulan with various natural polymers in wound dressings is attracting much attention due to its antioxidant, antimicrobial, and non-immunogenic characteristics [110]. For example, emulsion chitosan-oxidized pullulan hydrogels with loaded clove oil are synthesized by covalent and physical cross-linking. Clove oil was emulsified and stabilized in a chitosan solution and hardened by Schiff base covalent cross-linking with oxidized pullulan and thereafter subjected to freeze–thaw cycles for physical cross-linking (Figure 9) [111]. The resulting hydrogels showed strong radical scavenging (83%), antifungal, and antibacterial activity, with bacteriostatic activity after 2 and 3 days against *S. aureus* and *E. coli,* and can be applied as wound dressings.

**Figure 10:**
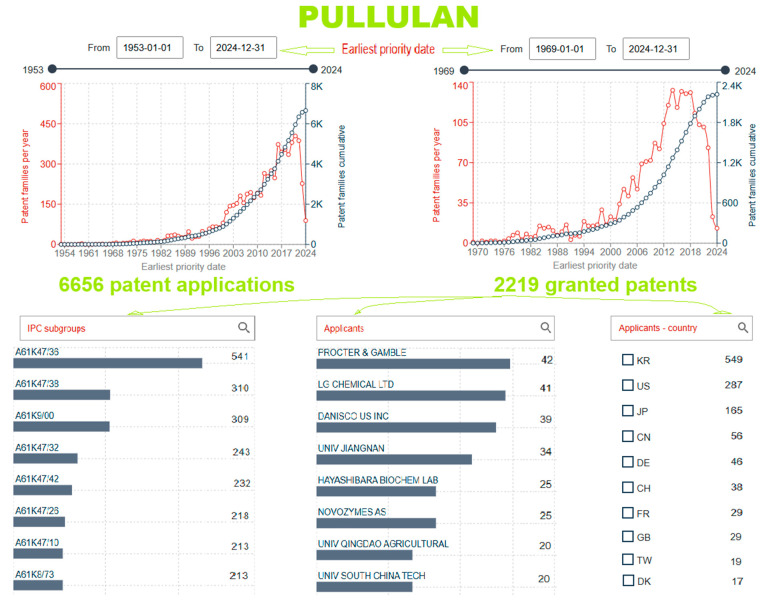
Pullulan: cumulative and yearly numbers of patent applications and granted patents depending on the earliest priority date (from 1953 to 2024), the IPC subgroups, the first eight applicants, and countries for granted patents. Two-letter codes: KR, US, JP, CN, DE, CH, FR, GB, TW, and DK are abbreviations for the following: Republic of Korea, United States of America, Japan, Republic of China, Germany, Switzerland, France, Great Britain, Taiwan Province of China, and Denmark, respectively. Data were obtained using the Espacenet database [14].

A patent literature survey for “pullulan” in the Espacenet database (according to the patent claims) shows that there are 6656 patent documents and 2219 granted patents from the first patent application in 1953 to the latest on 25 October 2024 (Figure 10). The country of origin of the first granted patent, DE1096850B, was Germany [112]. The patent search was additionally narrowed by adding keywords for specific fields: “pullulan” and ”gel” in the field title, “drug”, “carrier”, “deliver”, and “administration” only for granted patents. As a result of their visualization, the carefully chosen relevant granted patents are summarized in Table 4 and then analyzed.

Based on IPC subclasses, the largest number of registered patents is classified into the following classes:

- A61K47/36—Polysaccharides; derivatives thereof, e.g., dextrin (479);
- A61K9/00—Medicinal preparations characterized by special physical form (368);
- A61K47/38—Cellulose; derivatives thereof (322);
- A61K47/32—Macromolecular compounds obtained by reactions only involving carbon-to-carbon unsaturated bonds (240);
- A61K9/70—Medicinal preparations characterized by special physical form—web, sheet, or filament bases; films; fibres of the matrix type containing drug (199);
- A61K47/26—Medicinal preparations characterized by the non-active ingredients used, e.g., carriers or inert additives; targeting or modifying agents chemically bound to the active ingredient, organic compounds, carbohydrates, e.g., sugar alcohols, amino sugars, nucleic acids, mono-, di-, and oligo-saccharides; derivatives thereof (198).

Patent CN116420869B [113] discloses composite gel based on pullulan and ginkgo nut protein isolate, as well as the method of preparation. The prepared ginkgo nut protein isolate composite gel has good gel strength, and viscoelasticity is beneficial to promoting the development of the processing industry.

Invention CN116650436B [114] describes a novel gastric-soluble pullulan–nanocellulose composite hard capsule shell, the film-forming composition, and the process for preparation. Composite is obtained from high-acyl gellan gum as a gelling agent and nanocellulose as a filler, has the advantages of high humidity stability, high strength, easiness in disintegration in the stomach, and the like, and has important industrial application prospects. The anionic nanocellulose was obtained by high-pressure homogenization treatment on cellulose, with a length–diameter ratio greater than 100, a dispersity of less than 0.8, and a surface charge number greater than 20 mV.

An anti-adhesion composition in a film form of wound protection with excellent swelling properties and mucosal adhesion was the subject of granted patent KR102388509B1 [115]. This composition consists of a biocompatible mucoadhesive polymer and a specific swellable polymer that increases the barrier effect (pullulan, gelatin, hydroxypropylmethylcellulose, sodium carboxymethyl cellulose, methylcellulose, pectin, chitosan, starch, polyethylene oxide, polyacrylic acid, carrageenan, sodium alginate, jintan gum, and thereof combinations). An anti-adhesion agent is flexible and properly adheres to an application site by improving crumbling and adhesion, which is a problem with existing film-type anti-adhesion agents.

The method of preparing composite nanofiber based on pullulan–animal esterase protected by patent CN113355315B [116] includes the following phases: prepare an animal esterase solution by extracting and purifying animal liver, uniformly mixing a pullulan solution with obtained solution to form a pullulan–animal esterase electrostatic spinning solution, and finally carrying out electrostatic spinning to obtain the pullulan–animal esterase composite nanofiber. The animal esterase with an average diameter of 70.39–108.79 nm is uniformly distributed on the surface of the nanofiber. This method achieves immobilization of an animal esterase at nanomaterial and has the advantages of a simple process, low cost, environmental friendliness, and the like.

Granted patent KR102372384B1 [117] relates to an oral hygiene composition that is highly effective in suppressing, alleviating, or preventing bad breath and periodontal diseases. More precisely, this invention relates to an oral hygiene composition containing active ingredients: *Ginkgo biloba*, collagen, vitamin C, lysozyme chloride, a zinc compound, tocopherol, and *Allium sativum* extract.

The invention CN110384686B [118] relates to a method of preparing a sustained-release system drug (5-fluorouracil/mesoporous silica/oxidation pullulan) with pH response. The method of preparation includes the preparing steps of oxidation of pullulan, aminated mesoporous silica, 5-fluorouracil/aminated mesoporous silica, as well as the 5-fluorouracil/mesoporous silica/oxidation of pullulan drug sustained-release system. Oxidized pullulan and aminated mesoporous silica readily perform the Schiff base reaction. Pullulan has a good film-forming property, so drug-loaded aminated mesoporous silica can be coated to seal the pore channels. Controlled drug release can be carried out by simulating pH response due to the fact that acyl hydrazone bonds generated are sensitive to pH value. This release system has good biocompatibility and wide possibilities for application in biological medicine.

Patents CN114146046B [119] and JP7109675B2 [120] discloses a coated microneedle with a multilayer structure, including a base, a needle tip on the base, and a functional coating with a multilayer structure (a water-soluble polymer material, an active ingredient, and a sustained-release layer wrapping the content). A sustained-release layer is a time-delayed cross-linked sodium alginate (with a calcium source, gluconolactone, and a carrier) or a near-neutral chitosan system. The water-soluble polymer material is selected from the group consisting of pullulan, gelatin, hydroxypropyl methyl cellulose, carboxymethyl cellulose, hydroxyethyl cellulose, chitosan, and its derivatives, polyvinyl alcohol and its derivatives, polyvinylpyrrolidone and its derivatives, sodium hyaluronate, chondroitin sulfate, dextran and its derivatives, sodium alginate, one or more of polyacrylamide, glutamic acid, and polydopamine. The microneedles in these inventions are designed to prevent active ingredients from leaking at the application site, slow down the drug-containing matrix dissolution, and enhance drug absorption.

The inventions protected by CN108057122B [121] and CN106492225B [122] provide a drug delivery system based on pullulan with loaded adriamycin/doxorubicin for targeted administration on the liver. The methods of preparations comprise the subsequent steps: (1) a double-selenium compound preparation; (2) a carboxymethyl pullulan water solution and an EDC water solution mixture; adding dropwise DMF dissolved with the double-selenium compound into mixed liquid; adding an extracting agent into the reaction liquid; standing after the reaction is finished, taking subnatant; dialysis; freeze-drying and obtaining a pullulan carrier; (3) dissolving adriamycin hydrochloride and the pullulan carrier into DMSO (pullulan carrier: adriamycin = 2:1 *w*/*w*); mixing, dialysis, and ultrafiltration to obtain the pullulan drug carrying system. The stability of the drug delivery system is improved, and adriamycin/doxorubicin quantity is greatly increased.

Granted patent AU2018371143B2 [123] relates to a dosage form for an active ingredient to be dissolved in the oral cavity, comprising a first film layer and a second film layer that is arranged over the first film layer, wherein the composition of the first film layer can be identical to that of the second and comprises a water-soluble polymer, said first and the second film layers being interconnected by their overlapping edges so as to form at least one cavity and this cavity being filled with an active ingredient. In this configuration, the dosage form takes the form of a pouch made from two water-soluble film layers so that said film layers dissolve when the pouch is put in the mouth, and an active ingredient contained in said pouch can be released. This pouch configuration makes it possible to have a higher content of the active ingredient than comparable OTF films while avoiding thermal stress on said active ingredient during the production of the dosage form. The advantageous properties of known thin-film dosage forms are substantially retained. The invention also relates to a method for producing the dosage form.

A preparing method for hydrophilic three-dimensional nanofiber, applicable to tissue engineering and for cell growth, using electrospinning technology, as well as hydrophilic nanofiber prepared therefrom provides invention KR101828925B1 [124]. This method includes the phase of discharging a core part polymer solution (selected from the pullulan, gelatin, alginate, chitosan, hyaluronic acid, poly(gamma-glutamic acid), and polyvinyl alcohol); obtaining core–shell nanofiber; a core part hydrophilic polymer cross-linking; and finally, removing shell–part hydrophobic polymer.

Using an electric charge upset (realized from electronegativity to electropositive upset) according to invention CN105566511B [125], the rapid response of pullulan derivative (with important hepatoma/liver targeting character) can be formed to the weakly acidic tumor microenvironment and cellular inclusion/lysosome. A charge-overturning pullulan derivative in its molecular structure has a polyamino group and a pH-sensitive β-carboxylic acid amide-modified group (negatively charged under physiological conditions) and can be positive on the surface of the carrier. The process of its synthesis is simple under mild conditions. This charge-reversed pullulan derivative with high biosafety, biodegradability, good biocompatibility, nontoxicity, and no immunogenicity has significant advantages and extensive clinical application predictions in liver cancer targeted therapy.

The invention CN103961335B [126] protects a pullulan soft capsules and method for preparation. They were made by containing subsequent composition (weight percent): pullulan (20–60%), water (22–63%), plasticizer (15–30%), gel (0.15–2%), emulsifier (0.02–0.8%), and cosolvent (0.03–0.3%). The raw material of pure plant origin was used to avoid animal protein completely because of the pollution that it may cause. Pullulan polysaccharide soft capsule has impact strength, higher oxygen barrier, good quality stability, non-easy brittleness, and hydrolysis, and stabilized slaking index and surface for long-time placement do not emit fog. This capsule has a water content lower than a gelatin capsule and is more easily applicable to fill hygroscopic drugs and aldehyde-containing drugs, and it has market prospects and practical value widely.

Granted patent CN101555507B [127] describes the method for using pullulan in the preparation of maltotriose with high purity. The technological process comprises the following: (1) using an enzyme process with pullulanase to carry out zymohydrolysis on the pullulan; (2) using an ultrafiltration membrane to remove macromolecular proteins in reaction liquid; (3) removing substance-type micromolecular salt and condense the reaction liquid using a nanofiltration membrane; (4) chromatographic column separation to collect the pure product of the maltotriose and vacuum drying to obtain the finished product of the maltotriose over 99% purity and low cost. A great amount of pharmaceutical-grade maltotriose is used for fast diagnosis, curing acute pancreas diseases, and developing saccharides for use in medicine.

The polynuclear complex of iron(III) with oligomers of pullulan for treatment and prevention of syderopenic anemia was disclosed in invention EP1363951B1 [100].

The granted patents for pullulan-based drug carriers cover novel composite gels and methods for their preparation. They also disclose various administrations, e.g., stomach-soluble pullulan–nanocellulose composite hard shell capsules, pullulan soft capsules for filling hygroscopic drugs and aldehyde-containing drugs, anti-adhesion composition in the film form for wound protection, for the treatment of syderopenic anemia, oral hygiene composition for the treatment of periodontal diseases, for the sustained release of an anticancer drug with a pH response, coated microneedles with a multilayer structure for targeted administration to the liver. Using electrospinning technology, hydrophilic three-dimensional nanofibers have been applied in tissue engineering and for cell growth.

### 3.4. Pectin-Based Drug Carriers

**Figure 11:**
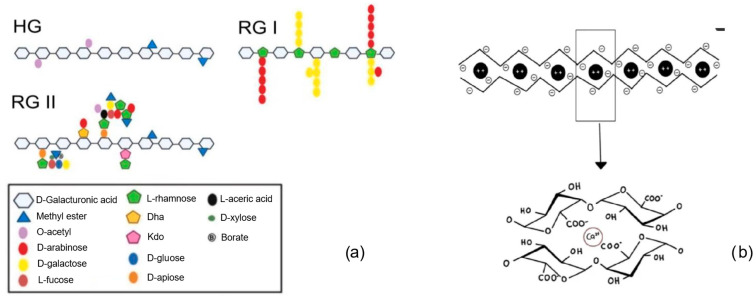
(**a**) Schematic diagram of various pectin structures: HG-homogalacturonan (the “smooth” region of α-(1,4)-linked D-galacturonic acid residues with methyl and acetyl esterification), RG-I—rhamnogalacturonan I (“hairy” region with alternating L-Rha and D-GalA residues and variable side chains) and RG-II—rhamnogalacturonan II (a complex structure containing up to 13 different sugars and 21 glycosidic linkages); (**b**) the “egg-box” model illustrating the gelling mechanism of low-methoxyl pectin. Reprinted from ref. [130] under open access creative commons CC-BY license.

Pectin is an anionic heteropolysaccharide, a structural polymer contained in the cell walls and primary and middle lamellas of terrestrial plants (from green parts). Henri Braconnot was the first to isolate pectin and defined it in 1825 [128]. Pectin consists of three main regions (Figure 11a): the “smooth” region is a homogalacturonan with linear chains of α-(1→4)-D-galacturonic acid and methylated carboxylic acid residues (e.g., D-xylose or D-apiose); “hairy” region of rhamnogalacturonan I (RG-I) with alternating D-GalA and L-Rha residues and variable side chains; and the third is rhamnogalacturonan II (RG-II) as a complex structure consisted of up to 13 various sugars with 21 glycosidic linkages [129,130]. Molecular weights of isolated pectins depend on plant species and isolation methods and vary between 28 kDa (apple pomace) and 753 kDa (sweet potato peels) [131]. The esterification degree of galacturonic acid residues is the highly critical parameter that affects gel-forming features and solubility of pectin [132]. Pectins are classified as high- or low-methoxy pectins (short HM-pectins or LM-pectins), with more or less than half of all the esterified galacturonic acid [133]. LM-pectins form gels by interacting with divalent cations, particularly Ca^2+^, as an idealized “egg box” model, wherein ionic bonds are formed between Ca^2+^ ions and the ionized galacturonic acid’s carboxyl groups at a pH value from 2.6 to 7.0 (Figure 11b) [130,134]. HM-pectins form a three-dimensional gel in acidic conditions (pH value 2.8–3.6) in the presence of a high sugar amount. It is cheap, biodegradable, biocompatible, water-soluble, and non-toxic, utilized with its modifications in many pharmaceutical [135] and biomedical applications [136]. Different types of pectin are applied in many drug delivery systems, e.g., as novel carriers for drugs in oral- [137] or colon-specific drug delivery [138,139] and cancer targeting [140].

**Figure 12:**
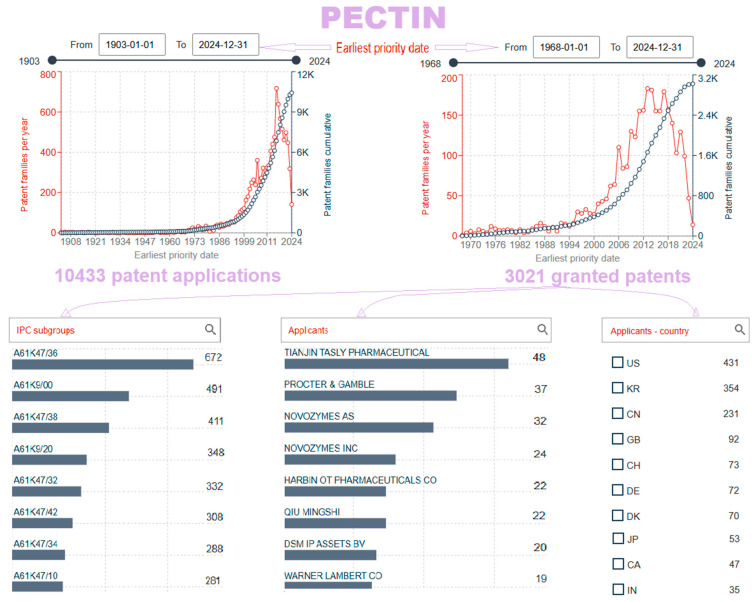
Pectin: cumulative and yearly numbers of patent applications and granted patents depending on the earliest priority date (from 1903 to 2024), the IPC subgroups, the first eight applicants, and countries for granted patents. Two-letter codes: US, KR, CN, GB, CH, DE, DK, JP, CA, IN, and are abbreviations for the following: United States of America, Republic of Korea, Republic of China, Great Britain, Switzerland, Germany, Denmark, Japan, Canada, and India, respectively. Data were obtained using the Espacenet database [14].

In the Espacenet database, a patent search for “pectin” by patent claims shows that there are 10433 patent documents and 3021 granted patents in the period from the first patent application in 1903 to the latest on 25 October 2024 (Figure 12). The first patent was granted in 1968, and the more intense growth of research and approved applications has been recorded since 2006. The country of origin of the first granted patent, DE1096850B, was Germany [141]. The search for patents was furthermore narrowed by additional keywords in the field title: “pectin”, ”gel”, “drug”, “carrier”, “deliver”, and “administration” only for granted patents. After their review, the attentively chosen relevant granted patents are shown in Table 5 and then shortly evaluated.

The great number of registered patents is classified into the following IPC subclasses:

- A61K47/36—Polysaccharides; derivatives thereof, e.g., dextrin (672);
- A61K9/00—Medicinal preparations characterized by special physical form (491);
- A61K47/38—Cellulose; derivatives thereof (411);
- Pills, tablets, discs, and rods (348);
- A61K47/32—Macromolecular compounds obtained by reactions only involving carbon-to-carbon unsaturated bonds (332);
- A61K47/34—Macromolecular compounds obtained otherwise than by reactions only involving carbon-to-carbon unsaturated bonds (288).

The invention CN117599195B [142] discloses a pectin ferrous slow-release iron supplement agent and its green preparation method, which includes the two green preparation processes. First, green extraction of a pectin raw material: only water is used as an extraction solvent, without any additives (which can cause degradation of the pectin molecules by residual acid and enzyme in the post-treatment). Second, pectin ferrous is obtained from a gel green process using only pectin, vitamin C, cane sugar, and ferrite. Due to pectin’s cross-linked structure, the iron agent and the vitamin C are slowly released in the stomach so that the large-scale release of the vitamin C and the iron agent, which can cause gastrointestinal tract stimulation, is avoided.

Piper betel pectin as well as preparation method and application thereof CN115746166B [143]. The invention belongs to the technical field of biological medicines and particularly relates to betel pectin as well as a preparation method and application thereof. The betel pectin is prepared by firstly pretreating betel with absolute ethyl alcohol, then heating it in a water bath with a sulfuric acid solution with stronger acidity for acid treatment, and finally, washing and purifying it multiple times with absolute ethyl alcohol so that the operation is simple; experiments prove that the obtained betel pectin can effectively protect oral mucosa epithelial cells from being damaged by betel nut components, enhance the activity of the oral mucosa epithelial cells, reduce the death of the oral mucosa epithelial cells, improve the morphology of the oral mucosa epithelial cells, enhance the barrier function of the oral epithelial cells and improve the mouth opening degree. The traditional Chinese medicine composition has a good effect on preventing and treating oral submucosal fibrosis.

The object of patent CN114711428B [144] is the application of composition based on pectin (e.g., citrus pectin) and (-)-epicatechin gallate in the inhibition of the pyridine derivatives formation in the gastrointestinal tract. According to invention, an antioxidant composition consists of pectin:(-)-epicatechin gallate in the mass ratio 1:1. Also, this combination has significant antioxidant capacity, stronger than (-)-epicatechin gallate or pectin alone, and it is used in the preparation of antioxidant health foods or medicines (associated with Alzheimer’s disease, diabetes, atherosclerosis, and others).

The method for preparation of wound dressing, based on composite hydrogel from shaddock peel pectin-oxidized chitosan, was described in patent CN114712553A [145]. It comprises the following steps: firstly, preparing shaddock peel pectin and oxidized chitosan, then preparing composite hydrogel, and finally, heating to obtain the wound dressing. The composite hydrogel prepared from the two materials (oxidized chitosan and shaddock peel pectin) has excellent biocompatibility, biodegradability, and renewability. It is a soft hydrogel, which can be easily removed from a wound skin without any secondary injury to the skin. It can be used in a healing process and as a nutrient; it is beneficial to cell proliferation and used for waste exchange. Wound dressing has a good treatment effect on large-area infections in wound surface and wound healing.

Methods for betaine-pseudo-phytate pectin gel, betaine-pseudo-acid berry pectin gel, and betaine-pseudo-uric acid catalpa pectin gel preparation were described in granted patent CN113893791B [146]. Obtained betanin-pseudo-acid berry pectin gels can be used to improve betanin stability. Betaine plays an important role in health care and medicine. Calcium chloride solution concentration and pH value in gel preparation have a greater impact on the betanin pigment stability.

Granted patent KR102636652B1 [147] protects pectin nanogel used for transdermal delivery of drug and method for its preparation. Pectin nanogel was obtained by modifying pectin by introducing a photo-crosslinkable functional group (norbornene), a photoinitiator, a crosslinker, and a bioactive substance, then sonication of pectin hydrogel (from 30 s to 5 min). Bioactive substances or drugs can be selected from hydrophobic or hydrophilic drugs, peptides, proteins, antibodies, fragments of antibodies, antibacterial agents, antigens, antibiotics, anticancer agents, anti-inflammatory agents, saccharides, or lipids. The polymerization method for obtaining nanogels is rapid by photo-cross-linking with ultraviolet radiation (wavelength 300 nm–500 nm, exposure time from 10 s to 15 min). Pectin nanogels have an average diameter of 10 nm to 1000 nm, possess excellent performance for skin penetration, and are suitable for use in cosmetics, medical devices, and pharmaceuticals.

A pectin- or gelatin-based antimicrobial surface coating material was protected by patent EP3494063B1 [148]. In this invention, boron compounds were mixed with pectin or gelatin to obtain a surface film-like coating material. The specified coating material can be applied in all packaging industries that require hygiene with antifungal, antibacterial, and anticandidal properties.

The method for preparing a novel pectin nano-drug based on multi-arm polyethylene glycol is the subject of granted patent CN105902520B [149]. This method exactly relates to a process in which eight-arm polyethylene glycol (8ARM-PEG-COOH) and pectin (PET) are carriers, and betulic acid and dihydroartemisinin are loaded. First, the 8ARM-PEG-COOH reacted with betulic acid and formed polyethyleneglycol-betulic acid (8ARM-PEG-BA), pectin reacted with dihydroartemisinin and formed pectin-dihydroartemisinin (PET-DHA). After that, PET-DHA and 8ARM-PEG-BA reacted and formed BA-PEG-PET-DHA. They are self-assembled and formed nanoparticles BA-PEG-PET-DHA (HCPT)·NPs with 10-hydroxycamptothecin to achieve strong targeting and high drug loading efficiency. The other method for preparing a nano-drug based on pectin-multi-arm polyethylene glycol over self-assembling is subject to granted patent CN105879052B [150]. By eight-arm polyethylene glycol (molecular weight 20,000) and pectin loaded with ursolic acid, connected by an ester bond, said self-assembling pectin-multi-arm polyethylene glycol nano-drug. Eight-arm polyethylene glycol can carry various hydrophobic or water-soluble drugs, e.g., the anticancer drug 10-hydroxycamptothecine is wrapped in the self-assembling process of nanoparticles. Obtained nano-drugs have good targeting capability (e.g., for sustained release and cancer targeting), good stability and biological degradability, low toxicity, and a controlled release function.

In patent CN104367588B [151], the drug carrier material is pectin, the hydrophobic retardation material is ethyl cellulose or an aqueous dispersion of ethyl cellulose, the gelling agent is zinc chloride or zinc acetate. They form a skeleton network of the composite gel. This improves colon-release drug delivery due to the hydrophobicity of the carrier material and its reduction of swelling properties. Once the pellets reach the colon, the carrier material is broken down by colonic plexus enzymes. The cumulative dexamethasone release in gastric and intestinal fluid was <20%, while in colonic fluid, the cumulative release was over 80% in vitro. The drug leakage problem in the upper end of the gastrointestinal tract is solved by a drug delivery system using polysaccharides. Zinc, as a significant trace element, is needed for damaged intestinal mucosa repair; pectin has a protective effect, which can reduce drugs’ irritating effect on the gastrointestinal mucosa, and is a probiotic source of intestinal flora.

An object of patent CN102940888B [152] is a method for obtaining pharmaceutical preparation based on a gel microsphere containing pectin/lecithin pellet for oral administration of colon-targeted drugs (ketoprofen, indomethacin, acemetacin, mesalamine, diclofenac sodium, or budesonide). First, pectin is mixed and dissolved in distilled water to form a gelatinous solution. Aqueous alkali is added for saponification, then stirred and mixed to make glue pectin solution. Second, lecithin is added to the pectin solution and mixed, and then the drug is added to form a uniform suspension by sonication. Third, the above-mentioned suspension is slowly dropped into 1–6% bivalent metal ion salt solution (calcium ions, zinc ions, hydrochloride salts of iron ions or sulfate salts) and performed a gel solidification reaction after dripping it is placed and filtered to form a gel ball. Fourth, washed with distilled water, obtained gel balls are dried at room temperature to obtain a finished product. The lecithin–pectin mass ratio is from 1:4 to 8:4.

Most of the analyzed granted patents for pectin-based gels cover different compositions, methods of their preparation, and various applications, such as gel microspheres for oral administration of colon-targeted drugs (ketoprofen, indomethacin, acemetacin, mesalamine, diclofenac sodium, or budesonide) or the prevention and treatment of oral submucosal fibrosis. A skeletal network of the composite gel enhances colon-release drug. Granted patents protect an antioxidant hydrogel composite applied for wound dressings, pectin nanogels for transdermal drug delivery (hydrophobic or hydrophilic drugs, peptides, proteins, antibodies, antibody fragments, antibacterial agents, antigens, antibiotics, anticancer agents, anti-inflammatory agents, saccharides, or lipids). Inventive examples include the application of biological medicines for preventing and treating oral submucosal fibrosis, an antioxidant composition, pseudo-acid berry pectin gels for improving betanin stability, a pectin-based antimicrobial, antifungal, anticandidal surface coating material.

## 4. Conclusions and Future Trends

This paper provides a systematized survey of the latest published documents (articles and especially patent applications and granted patents) considering innovations in the field of natural polysaccharides: fucoidan, pullulan, dextran, and pectin. This review, based on polysaccharide-based gels for drug delivery applications, highlights their significance due to their unique functional groups, easy gelling ability, simple modifications, biodegradability, biocompatibility, nontoxicity, and usually low cost. The description of analyzed polysaccharides accents their individual features applied to build novel drug delivery systems. Numerous investigations published in scientific papers, books, and patent documents show that polysaccharide gels and their derivatives have better drug delivery efficiency and are viable candidates for numerous pharmaceutical and medical applications. This review can serve as a better assessment for conducting future research in order to produce commercial formulations of therapeutic agents or improved drug carriers. Patents not only contain unique information but also show innovative applications of polysaccharide-based drug delivery systems and new research trends.

Future trends in polysaccharide-based drug carriers may include newly granted patents aimed at improving encapsulated drug protection and delivery efficiency by overcoming biological barriers. New inventive solutions may be directed toward the development of self-healing polysaccharide-based gels applied in tissue engineering and regenerative medicine. Further developments may include novel stimulus-sensitive gels, which change properties in response to environmental changes (i.e., pH, temperature, light) as safe carriers for modified drug release. As natural, sustainable, and biodegradable resources, polysaccharides may significantly replace synthetic ones and be the basis for the development of novel composite biomaterials with improved performance. Advanced manufacturing technology (using the electrospinning method and 3D bioprinting) provides new opportunities for complex structure production in the pharmaceutical industry.

Certain limitations of the polysaccharide gel application include variability in the composition of natural sources (algae, plants) due to different cultivation and extraction conditions, and their modification is often complex and expensive. Some modifications can trigger immune reactions, and it is also difficult to precisely regulate the degradation rate, which is crucial for applications such as drug delivery or implants. Natural polysaccharides are susceptible to microbial spoilage and therefore require preservation. Polysaccharide gels can be more expensive than synthetic ones due to more complex production and extraction processes.

This overview may be useful in new investigations and as an inspiration for future studies in the field of polysaccharide-based drug carriers.

## Figures and Tables

**Table 1:** List of IPC codes used for the polysaccharide-based drug delivery systems [16,17].

IPC Code	IPC Description as Indicated in the Relevant CPC Symbol
A61K	Preparations for medical, dental, or toiletry purposes (devices or methods specially adapted for bringing pharmaceutical products into particular physical or administering forms A61J3/00; chemical aspects of, or use of materials for deodorization of air, for disinfection or sterilization, or for bandages, dressings, absorbent pads, or surgical articles A61L; soap compositions C11D)
A61K9/00	Medicinal preparations characterized by special physical form (nuclear magnetic resonance contrast preparations or magnetic resonance imaging contrast preparations A61K49/18; preparations containing radioactive substances A61K51/12)
A61K9/06	Ointments; bases thereof; other semi-solid forms, e.g., creams, sticks, gels (composition of ointments, creams, or gels A61K47/00)
A61K31/00	Medicinal preparations containing organic active ingredients
A61K31/70	• Carbohydrates; sugars; derivatives thereof
A61K31/715	•• Polysaccharides, i.e., having more than five saccharide radicals attached to each other by glycosidic linkages; derivatives thereof, e.g., ethers, esters
A61K31/737	••• Sulfated polysaccharides, e.g., chondroitin sulfate, dermatan sulfate
A61K8/18 A61K8/72 A61K8/73	Cosmetics or similar toiletry preparations • Characterized by the composition •• Containing organic macromolecular compounds ••• Polysaccharides
A61P35/00	Antineoplastic agents
A61K43/00	Drugs for specific purposes, not provided for in groups A61P1/00-A61P41/00
A61K47/00	Medicinal preparations characterized by the non-active ingredients used, e.g., carriers or inert additives; targeting or modifying agents chemically bound to the active ingredient
A61K47/06	• Organic compounds, e.g., natural or synthetic hydrocarbons, polyolefins, mineral oil, petrolatum, or ozokerite
A61K47/08	•• Containing oxygen, e.g., ethers, acetals, ketones, quinones, aldehydes, peroxides
A61K47/10	••• Alcohols; phenols; salts thereof, e.g., glycerol; polyethylene glycols; poloxamers; PEG/POE alkyl ethers
A61K47/30	• Macromolecular organic or inorganic compounds, e.g., inorganic polyphosphates
A61K47/32	•• Macromolecular compounds obtained by reactions only involving carbon-to-carbon unsaturated bonds, e.g., carbomers, poly(meth)acrylates, or polyvinyl pyrrolidone
A61K47/34	•• Macromolecular compounds obtained other than by reactions only involving carbon-to-carbon unsaturated bonds, e.g., polyesters, polyamino acids, polysiloxanes, polyphosphazines, copolymers of polyalkylene glycol, or poloxamers (…)
A61K47/36	••• Polysaccharides; derivatives thereof, e.g., gums, starch, alginate, dextrin, hyaluronic acid, chitosan, inulin, agar, or pectin
A61K47/38	Cellulose; derivatives thereof
A61P29/00	Non-central analgesic, antipyretic or anti-inflammatory agents, e.g., antirheumatic agents; non-steroidal anti-inflammatory drugs [NSAIDs]
C08B37/00	Preparation of polysaccharides not provided for in groups C08B1/00–C08B35/00; derivatives thereof (cellulose D21; microbiological processes C12P)

**Table 2:** Selected relevant patents of fucoidan-based drug carriers.

Patent Title	Publication No. [Reference]	Applicant/s	Earliest Priority
Anti-wrinkle micro-emulsion gel eye patch and preparation method thereof	CN118319827B [50]	Karuiji Beijing Life Tech Co., Ltd.; Beijing Yiguantang Biotechnology Co., Ltd.	13 June 2024
Brown algae polysaccharide as well as preparationmethod and application thereof	CN117247472B [51]	Univ. Guangdong Medical; Guangdong Zhanjiang Marine Medicine Res Inst.	14 September 2023
Gastric retention tablet and preparation method thereof	CN116077417B [52]	By-Health Co., Ltd.	29 December 2022
Microbead for embolization and composition for treatment of proliferative diseases	KR102386631B1 [53]	P.L Micromed Co., Ltd.	9 April 2021
CuET-coated Fuc nano drug delivery system based on selectin targeting, preparation method and application of CuET-coated Fuc nano drug delivery system in antitumor drugs	CN112999361B [54]	Nanjing University of Technology	18 March 2021
Anticoagulant fucoidan oligosaccharide and preparation method thereof	CN112521431B [55]	Biology Institute of Shandong Academy of Sciences	10 December 2020
Fucoidan composition and use thereof	TWI747567B [56]	National Taiwan Ocean University	22 October 2020
Composite antibacterial gel as well as preparation method and application thereof	CN111888430B [57]	Shenzhen Yinghansi Tech Co., Ltd.	14 August 2020
Preparation method and application of shear-responsivenano drug delivery system	CN111840527B [58]	Univ Zhengzhou A	15 June 2020
Hemostatic gel as well as preparation method and application thereof	CN111760066B [59]	Beijing University of Chemical Technology	9 June 2020
A method for differentiation of mesenchymal stem cells from pluripotent stem cells	KR102275454B1 [60]	Konkuk University Industrial Cooperation Co.	7 May 2020
Preparation method and application of fucoidan-coated gated mesoporous manganese dioxide nano drug-loading system	CN111298133B [61]	Zhengzhou University	2 March 2020
Personal care compositions and methods for the same	US11540980B2 [62]	Colgate-Palmolive Company	20 December 2019
Novel sea cucumber fucoidan and preparation method and application thereof	CN110437288B [63]	Ocean Universityof China	2 September 2019
Voglibose tablet and preparation method thereof	CN110101671B [64]	Jiangsu Chenpai Pharmaceutical Group Corp.	11 April 2019
Preparation method and application of Sargassum henslowianum fucosan sulfate	CN111748045B [65]	Tianjin Medical University	27 March 2019
Pharmaceutical delivery compositions and uses thereof	GB2595109B [66]	Innovacorium, Inc.	10 January 2019
Highly purified fucans for the treatment of fibrousadhesions	AU2019310194B2 [67]	ARC Medical Devices Inc.	2 November 2018
Low endotoxin fucan compositions, systems and methods	AU2019310360B2 [68]	ARC Medical Devices Inc.	1 August 2018
Highly purified and/or modified fucan compositions for the treatment of fibrous adhesions	ZA202100275B [69]	ARC Medical Devices Inc.	2 November 2018
Fabrication and characterization of core-shell nanofiberPCL@gelatin/fucoidan by electrospinning	KR101973806B1 [70]	Hannam University Institute for Industry-Academia Cooperation	29 November 2017
Biocompatible nanoparticle and use thereof	KR101893549B1 [71]	Kyungpook National University Industry-Academic Cooperation Foundation	20 January 2016
Oral dissolving film for achieving fast onset of action,enhancing medicine availability, fully developing therapeutic effect and reducing side effects	TWI612978B [72]	Eh Seng Pharmaceutical Mfg. Co., Ltd.	26 August 2015
Low-molecular-weight fucoidan and effect thereof on diabetic nephropathy	CN103804504B [73]	Shandong University, Weihai	22 January 2014
Scaffold containing drug delivery system	KR101239782B1 [74]	Industry-Academic Cooperation Foundation, Chosun University	28 November 2011

**Table 3:** Selected relevant patents of dextran-based drug carriers.

Patent Title	Publication No. [Reference]	Applicant/s	Earliest Priority
ROS responsive polymer Mal-PHB-dextran and cell knapsack drug delivery system	CN115594776B [90]	Shandong University	19 September 2022
Amphiphilic dextran derivative carrier targeting tumor-associated fibroblasts and preparation and application of pharmaceutical composition of amphiphilic dextran derivative carrier	CN112876578B [91]	China Pharmaceutical University	21 January 2021
Difunctional dextranase with chitosan hydrolytic activity, gene, carrier, engineering strain and application of difunctional dextranase	CN106978410B [92]	Pray Bio-Technology (Mingguang) Co., Ltd.	31 March 2017
Method for preparing antitumor pH-sensitive and non-pH-sensitive dextran-polylactic acid-polyethylene glycol nanoparticle drug delivery system	CN106860875B [93]	Nanjing Nuoyuan Medical Equipment Co., Ltd.	7 March 2017
Dextran magnetic iron nanoparticle, preparation method and use thereof for cancer comprising a dextran layer, a magnetic iron nanoparticle and an active drug, and useful for the treatment of cancer and for imaging	TWI644687B [94]	Sagabio Co., Ltd.	30 November 2016
Dextran cross-linked hemoglobin-based oxygen carrier, preparation method thereof and application	CN106421805B [95]	Institute of Transfusion Medicine, The Academy of Military Medical Sciences	14 September 2016
Photo-crosslinkable dextran polymer drug delivery withionic functional group and acrylate group and method for preparing the same	KR101842059B1 [96]	INHA University Research and Business Foundation	5 February 2016
Dextran nano-drug carrier, preparation method thereof,anti-tumor drug and preparation method thereof	CN105148279B [97]	The Second People’s Hospital of Shenzhen	26 June 2015
Biodegradable microbeads with improved anticancer drug adsorptivity, containing albumin and dextran sulfate, and preparation method therefor	EP2921166B1 [98]	Utah-Inha DDS & Advanced Therapeutics Research Center	15 November 2012
Benefit agent delivery particles comprising dextran	EP2747741B1 [99]	Unilever Plc; Unilever N.V.	24 August 2011

**Table 4:** Selected relevant patents of pullulan-based drug carriers.

Patent Title	Publication No. [Reference]	Applicant/s	Earliest Priority
Pullulan and ginkgo nut protein isolate composite gel and preparation method thereof	CN116420869B [113]	Nanjing Forestry Univ; Baima Future Food Res. Inst.	6 April 2023
Gastric-soluble pullulan–nanocellulose composite hard capsule shell and preparation process thereof	CN116650436B [114]	Ocean University of China	17 March 2023
Film-type anti-adhesion composition with excellent mucosal adhesion and swelling properties	KR102388509B1 [115]	CNLD Co., Ltd.; Industry-Academic Cooperation Found., Yonsei University	14 June 2021
Preparation method of pullulan–animal esterase composite nanofiber	CN113355315B [116]	Yangzhou University	31 May 2021
Oral hygiene composition for preventing or alleviating periodontal disease and halitosis	KR102372384B1 [117]	Nutrabbit Co., Ltd.	16 July 2020
Preparation method of 5-fluorouracil/mesoporous silica/oxidation pullulan drug sustained-release system with pH response	CN110384686B [118]	Changzhou University	2 July 2019
Coating microneedle with multilayer structure, preparation method of coating microneedle and microneedle patch comprising coating microneedle	CN114146046B [119]	Technical Institute ofPhysics and Chemistry of the Chinese Academy of Sci.	31 August 2018
Implantable slow-release microneedle patch and preparation method thereof	CN110917176B JP7109675B2 [120]	Zhongke Microneedle (Beijing) Technology Co., Ltd.	31 August 2018
Natural pullulan polysaccharide medicine carrying system loaded with adriamycin and preparation method thereof	CN108057122B [121]	Chongqing MedicalUniversity	11 February 2018
Pullulan and all-transretinoic acid drug carrier system and preparation method thereof	CN106492225B [122]	Chongqing MedicalUniversity	23 November 2016
Pouch-type orally dissolving films with high active ingredient concentration	AU2018371143B2 [123]	LTS LohmannTherapie-Systeme Ag	21 November 2017
Low-density nanofiber, a process for the preparation thereof, and use thereof	KR101828925B1 [124]	KNU-Industry Cooperation Foundation	12 October 2016
Charge reversal pullulan derivative and synthesis method and application thereof	CN105566511B [125]	Tianjin Medical University	27 January 2016
Pullulan soft capsule and preparation method thereof	CN103961335B [126]	Shandong JinmeiBiotechnology Co., Ltd.	22 May 2014
Method for using pullulan to prepare high-purity maltotriose	CN101555507B [127]	Yangling Yizhinong Microorganism Eng. Inst. Co., Ltd.	18 May 2009
Polynuclear complex Fe(III) with pullulan oligomers,process of its obtaining and pharmaceutical preparations on the basis of the complex	EP1363951B1 [100]	Zdravlje AD	7 December 2001

**Table 5:** Selected relevant patents of pectin-based drug carriers.

Patent Title	Publication No. [Reference]	Applicant/s	Earliest Priority
Ferrous pectin slow-release iron supplement agent and green preparation method thereof	CN117599195B [142]	Guangdong Pharmaceutical University	29 November 2023
Piper betel pectin as well as preparation method and application thereof	CN115746166B [143]	Hainan University	1 December 2022
Application of pectin ECG composition in inhibition of formation of gastrointestinal pyridine derivatives	CN114711428B [144]	Hubei University of Technology	11 April 2022
Preparation method of wound dressing based on shaddock peel pectin-oxidized chitosan composite hydrogel	CN114712553A [145]	Zhejiang Sci-Tech University	11 April 2022
Preparation method of betanin-Nicandra physaloides stillingia pectin gel	CN113893791B [146]	Guangxi Academy of Agricultural Sci. Nanchang University	8 October 2021
Pectin nanogel for transdermal delivery and preparation method thereof	KR102636652B1 [147]	Seoul National University R&Db Foundation	5 April 2021
Pectin- or gelatin-based antimicrobial coating	EP3494063B1 [148]	Yeditepe Universitesi	5 August 2016
Method for preparing nano-drug common delivery system based on pectin and multi-arm polyethylene glycol	CN105902520B [149]	Beijing Forestry University	13 June 2016
Method for preparing nano-drug through self-assembling of pectin-multi-arm polyethylene glycol	CN105879052B [150]	Beijing Forestry University	6 June 2016
Dexamethasone, pectin and zinc combined gel oral colon-specific drug delivery pellet	CN104367588B [151]	Shanxi Medical University	29 November 2014
Targeted drug delivery method of pectin colon containing lecithin	CN102940888B [152]	Tianjin University of Science & Technology	12 November 2012

## Data Availability

The data presented in this study are available in [https://www.mdpi.com/2073-4360/16/19/2834] at [https://doi.org/10.3390/polym16192834], reference number [87]. The data presented in this study are available in [https://www.mdpi.com/2310-2861/10/4/227] at [https://doi.org/10.3390/gels10040227], reference number [111]. The data were derived from the following resources available in the public domain: [https://worldwide.espacenet.com/]. [Figure 6] [https://doi.org/10.3390/polym16192834] [87]. [Figure 9] [https://doi.org/10.3390/gels10040227] [111].
